# Interpretable Feature-Transformer Framework for Cross-Subject MCI Detection Using Nonlinear Dynamical and Graph-Theoretic EEG Features

**DOI:** 10.21203/rs.3.rs-8744978/v1

**Published:** 2026-02-11

**Authors:** Hadi Azizpour Lindi, Reza Shalbaf, Ahmad Shalbaf, Mohsen Sadat Shahabi, Peyman Abharian

**Affiliations:** 1 Cognitive Modeling, Institute for Cognitive Science Studies, Chamran, Pardis, 1658344575, Tehran, Iran.; 2 Biomedical Engineering, Shahid Beheshti University of Medical Sciences, Velenjak, Tehran, Tehran, Iran.

**Keywords:** MCI, Graph Theory, Nonlinear Dynamics, Entropy, Transformer, EEG, Deep Learning, Alzheimer’s disease, EEGNet, SHAP, Resting State

## Abstract

Early and accurate detection of Mild Cognitive Impairment (MCI) is essential for preventing progression toward Alzheimer’s disease (AD). In this cross-subject study, we investigate the effectiveness of entropy- and graph-based EEG features for distinguishing MCI from healthy controls (HC), using two modeling approaches: (1) a Transformer network applied to the engineered feature set, and (2) an EEGNet model trained on the same feature representation for comparison. The dataset consists of resting-state, eyes-closed EEG recordings from 183 participants (127 HC, 56 MCI), collected using a 20-channel STAT^™^ X24 wireless system and segmented into 3-second epochs. EEG data underwent standard preprocessing, including band-pass filtering, downsampling, normalization, and class-balancing augmentation applied to the minority class. From each channel, nonlinear dynamical measures (e.g., sample and fuzzy entropy, Higuchi fractal dimension, Lyapunov exponent) and graph-theoretic connectivity descriptors derived from coherence matrices across five frequency bands were extracted, yielding a structured 19×77 feature representation. The feature-based Transformer achieved the best performance (97.04% ± 0.72), outperforming the feature-based EEGNet baseline and highlighting the benefits of combining rich handcrafted features with attention-based modeling. SHAP (SHapley Additive exPlanations) analysis provided global and local interpretability, revealing the most influential nonlinear and connectivity features as well as the EEG channels contributing most to classification. Overall, these results demonstrate the effectiveness of feature-Transformer integration and support the potential of interpretable feature-driven deep learning models for early MCI detection.

## 1 Introduction

Cognitive decline constitutes one of the most significant global health challenges of the twenty-first century, affecting tens of millions of individuals worldwide [1]. Among its primary causes, Alzheimer’s disease (AD) is a progressive neurodegenerative disorder accounting for approximately 60–80% of all dementia cases [2]. AD predominantly affects older adults and is characterized by a prolonged preclinical phase during which neuropathological changes accumulate for years or even decades before the emergence of overt clinical symptoms [2]. Situated between normal cognitive aging and dementia is a transitional clinical condition known as Mild Cognitive Impairment (MCI). In particular, amnestic Mild Cognitive Impairment (aMCI) is defined by measurable cognitive decline, most notably in memory, that exceeds age-related changes while largely preserving independence in daily functioning [3]. MCI is widely recognized as a prodromal stage of AD, with an estimated annual conversion rate of 10–15% [4]. Consequently, the development of reliable methods for early and accurate MCI detection is critical for enabling timely interventions and potential disease-modifying strategies. Over the past decade, diagnostic approaches to AD and MCI have shifted from symptom-based assessments toward biologically grounded frameworks that emphasize underlying neuropathological processes [2]. This paradigm shift is formalized in the AT(N) framework, which categorizes biomarkers according to amyloid pathology (A), tau pathology (T), and neurodegeneration (N). Although neuroimaging modalities such as positron emission tomography (PET) and functional magnetic resonance imaging (fMRI) provide valuable insights into these biological mechanisms, their high cost, limited accessibility, and invasive nature restrict their suitability for large-scale screening and longitudinal monitoring. In contrast, resting-state electroencephalography (EEG) offers a non-invasive, cost-effective, and widely accessible alternative for assessing brain function, providing direct measurements of electrophysiological activity with high temporal resolution. EEG is particularly well suited for studying cognitive decline, as it reflects both local neural oscillations and large-scale network dynamics underlying cognitive processing. Numerous studies have reported characteristic EEG alterations in MCI and AD, including increased spectral power and coherence in low-frequency bands (delta and theta) alongside reduced alpha and beta activity [5, 6]. These patterns are commonly interpreted as cortical slowing and impaired functional integration among distributed neural assemblies. Recent advances in portable EEG systems and high-density recordings have further strengthened EEG’s potential as a scalable biomarker for early AD detection [4, 7].

Despite these advantages, EEG analysis remains challenging due to the high dimensionality, non-stationarity, and susceptibility of EEG signals to noise and artifacts. Traditional machine learning (ML) approaches, such as support vector machines (SVMs) [8], k-nearest neighbors (kNN), and decision trees [9], have achieved moderate success but rely heavily on handcrafted features derived from domain expertise. Such manual feature engineering is often labor-intensive and may inadequately capture the nonlinear, dynamic, and multiscale nature of brain activity. To address these limitations, deep learning (DL) methods have emerged as powerful alternatives capable of learning hierarchical representations directly from EEG data. Among DL architectures, convolutional neural networks (CNNs) have been widely adopted for EEG analysis due to their ability to learn spatial and temporal filters from multichannel signals. A prominent example is EEGNet, a compact and computationally efficient CNN architecture specifically designed for EEG-based applications [10]. EEGNet employs depthwise and separable convolutions to explicitly model temporal dynamics and spatial channel interactions while maintaining a lightweight parameterization and strong generalization capability. As a result, EEGNet has become a widely accepted baseline in EEG research. Recent extensions have further demonstrated its flexibility, including spatiotemporal 3D variants that enhance interpretability through saliency- and permutation-based analyses [11], as well as quantized implementations optimized for edge computing that significantly reduce memory usage and energy consumption with minimal performance degradation [12]. However, CNN-based architectures such as EEGNet primarily rely on local receptive fields and hierarchical feature stacking, which limits their ability to explicitly model long-range temporal dependencies and global cross-channel interactions. Recurrent neural networks (RNNs) and long short-term memory (LSTM) models were introduced to address temporal modeling in EEG [13], and hybrid CNN–LSTM architectures have demonstrated improved performance by jointly encoding spatial and temporal information [14–16]. Nonetheless, these sequential models are constrained by vanishing gradient issues, limited parallelization, and inefficiencies in modeling long-range dependencies. The introduction of attention mechanisms marked a major shift in sequence modeling by enabling neural networks to dynamically focus on the most informative components of an input. Building upon this concept, the Transformer architecture relies entirely on multi-head self-attention to model global dependencies without recurrence [17]. Although originally developed for natural language processing, Transformers have recently demonstrated strong performance in EEG analysis. Studies have shown that Transformer-based models applied directly to raw EEG signals outperform traditional handcrafted feature pipelines [18], while hybrid convolutional–Transformer architectures improve joint temporal–spatial modeling in cognitive and motor imagery tasks [19]. A recent systematic review further highlights the growing adoption of Transformers in EEG decoding and emphasizes their capacity to naturally capture spatial, temporal, and channel-wise dependencies within a unified framework [20]. These characteristics make attention-based architectures particularly well suited for modeling the distributed and dynamic neural processes associated with MCI.

Beyond end-to-end learning from raw EEG, a substantial body of prior work has emphasized the diagnostic value of handcrafted features grounded in neurophysiology. Graph-theoretic approaches model EEG electrodes as nodes in functional brain networks, with topological measures such as clustering coefficient, characteristic path length, and small-worldness quantifying network efficiency and integration [21]. Disruptions in these metrics have been consistently reported in MCI and AD populations [22, 23]. Complementarily, nonlinear dynamical analyses characterize signal complexity and irregularity, which tend to be reduced in neurodegenerative conditions [24]. Measures such as correlation dimension, Lyapunov exponents, entropy-based metrics, and fractal dimensions have demonstrated strong discriminative power between healthy aging, MCI, and AD [25–27]. Recent hybrid approaches combining handcrafted features with deep learning models have further improved classification performance and interpretability, suggesting that engineered and learned representations capture complementary aspects of brain function [28, 29]. Nevertheless, a major barrier to the clinical translation of DL-based EEG models remains their limited interpretability. Explainable artificial intelligence (XAI) techniques, particularly SHAP (SHapley Additive exPlanations), offer a principled, game-theoretic framework for attributing model predictions to individual features [30]. In EEG-based dementia research, SHAP has been shown to uncover clinically meaningful biomarkers while enhancing transparency in CNN- and Transformer-based models [31–34].

Most existing EEG-based MCI studies rely either on handcrafted features combined with conventional classifiers or on end-to-end deep learning models trained on raw signals-approaches that often suffer from limited cross-subject generalization and reduced interpretability. Moreover, prior deep learning methods rarely integrate structured nonlinear or graph-theoretic descriptors within attention-based frameworks. To address these limitations, this study proposes a feature-driven Transformer architecture that leverages entropy-based, nonlinear dynamical, and graph-theoretic EEG representations while incorporating SHAP-based interpretability. By unifying structured neurophysiological descriptors with attention mechanisms, the proposed approach aims to provide a robust, generalizable, and clinically meaningful framework for early MCI detection.

## 2 Methods

### 2.1 Dataset

This study employed EEG data obtained from the dataset reported by Meghdadi et al. [5, 35]. Although the original dataset included participants aged 18 to 90 years, the data provided to us encompassed only individuals within the 40–90-year age range. The final sample consisted of 183 participants, including 56 individuals with MCI and 127 healthy controls (HC). Among these, 98 were male and 85 were female, with the gender distribution across diagnostic groups summarized in Table 1.

The data were collected from multiple research centers specializing in AD and cognitive aging. Individuals with MCI were identified and enrolled through different recruitment and diagnostic procedures across these centers. Notably, in one of these institutions, individuals with aMCI were specifically targeted, representing more than half of the MCI group.

Participants were recruited from four research centers in the United States:

1. Advanced Brain Monitoring (ABM), Carlsbad, California
2. Advanced Neurobehavioral Health (ANH), San Diego, California
3. Massachusetts General Hospital (MGH), Boston, Massachusetts
4. Mayo Clinic (MAYO), Rochester, Minnesota

Due to ongoing EEG research, the total number of participants increased compared to the original publications. Specifically, in the data provided by ABM, there were 16 additional individuals beyond those reported in A. H. Meghdadi et al. [5], including three MCI and thirteen healthy controls, all aged over 40. At ABM (Carlsbad, CA), healthy aging participants were recruited through online advertisements and flyers. These individuals were cognitively normal adults aged 40–90 years, forming the HC group. Participants diagnosed with MCI were recruited from MGH, ANH, and MAYO, where diagnostic procedures differed slightly between centers. At ANH, individuals with amnestic MCI were identified according to DSM-5 criteria for Minor Neurocognitive Disorder (MCI). Diagnosis required measurable memory impairment (≥ 1.5 SD below the normative mean), absence of decline in daily functioning, and no medical or psychiatric condition explaining cognitive symptoms. Functional independence was confirmed by an informant, typically a spouse or adult child. At MGH, participants were drawn from a longitudinal cohort as part of a study on brain aging and cognition. Recruitment sources included affiliated hospitals and community advertisements. Participants were neurologically and psychiatrically healthy based on clinical screening and exhibited normal global cognition (MMSE: 24–30). MCI diagnosis followed the comprehensive neuropsychological criteria proposed by Jak et al. [36], which required at least two cognitive domains to show two or more test scores one standard deviation below the normative mean. All participants underwent a neuropsychological test battery consisting of 14 standardized assessments evaluating global cognition, premorbid intelligence, and four specific cognitive domains. Test scores were demographically corrected for age, sex, and education. Neuropsychological classification was based on sixteen standardized performance scores derived from ten tests across the four domains.

EEG and ECG recordings were obtained using the STAT^™^ X24 system (Advanced Brain Monitoring, Carlsbad, CA), an FDA-cleared wireless EEG device. EEG signals were recorded from 20 scalp channels arranged according to the 10–20 montage and referenced to linked mastoids. An additional ECG channel was recorded from sensors placed on the right and left clavicles (ECG data were not included in this study). The STAT^™^ X24 system uses passive Ag/AgCl electrodes connected via flexible printed circuit cables on polyester strips. Data were sampled at 256 Hz, with amplifier bandpass filters of 0.1–100 Hz. Conductive cream (Kustomer Kinetics, Arcadia, CA) was used to ensure stable electrode contact, and impedance was maintained below 40kΩ, following manufacturer recommendations [37]. Recordings were conducted during a resting-state, eyes-closed condition, with participants instructed to remain awake. Each resting-state EEG recording lasted approximately 300 seconds (5 minutes). Before and after the eyes-closed session, participants also completed resting-state eyes-open recordings and three event-related potential (ERP) tasks, which are not analyzed in the present study.

### 2.2 Preprocessing and Data Preparing

All EEG signals underwent a standardized preprocessing and data preparation pipeline to ensure data quality and comparability across participants. Independent Component Analysis (ICA) was not applied in this study to avoid potential signal distortion and unnecessary complexity that could affect classification performance. Similarly, badchannel removal was not required, as the signals did not contain severely corrupted channels; after filtering, channel quality was deemed acceptable for analysis. Instead, an average reference was applied to all channels to re-reference the data. The continuous EEG recordings were first band-pass filtered between 0.5–40 Hz to remove slow drifts and high-frequency noise, then downsampled to 100 Hz. Each recording was segmented into non-overlapping 3-second epochs. From the original 20 recorded electrodes, the POz channel was excluded, leaving 19 standard channels for subsequent analysis. Each 3-second segment (19 channels × 300 samples) was treated as an independent input sample for classification. To minimize inter- and intra-subject variability, two levels of normalization were applied. First, channel-wise z-score normalization was performed within each subject, applied independently across time for each electrode. Second, subject-level normalization was applied across all epochs to align feature distributions between participants. To mitigate class imbalance, offline data augmentation was applied exclusively to the minority class (MCI). Augmentation consisted of Gaussian noise injection, amplitude scaling, and temporal shifting, which simulate physiologically plausible intra-subject variability without altering class labels. Augmented samples preserved the original subject identifiers, and all experiments employed subject-wise data partitioning using group-based splitting. Consequently, no subject, original or augmented, appeared in more than one data partition, ensuring strict cross-subject evaluation. Before augmentation, the preprocessed dataset comprised 17,894 epochs with a shape of (17894, 19, 300), corresponding to 183 subjects (127 HC and 56 MCI), each contributing approximately 98 epochs on average. A total of 40 epochs were discarded during preprocessing due to artifact contamination or filtering constraints. After augmentation, the dataset was balanced to include 12,410 MCI and 12,411 HC epochs, yielding a total of 24,821 samples with a final shape of (24821, 19, 300).

### 2.3 Feature Extraction

Feature extraction is a key step in converting EEG signals into informative representations for classification. In this study, we do not use raw EEG epochs as model input; instead, we rely exclusively on engineered features. Each of the 19 EEG channels was characterized using a comprehensive set of nonlinear dynamical descriptors and graph-theoretical connectivity measures designed to capture the complexity and network structure of brain activity. Specifically, 12 nonlinear features were extracted per channel to quantify signal irregularity, complexity, and fractal properties. In addition, functional connectivity matrices were computed across five canonical frequency bands, from which six local metrics (degree, weighted degree, clustering coefficient, between-ness centrality, eigenvector centrality, local efficiency) and seven global metrics (global efficiency, characteristic path length, average clustering coefficient, small-worldness, assortativity, network density, transitivity) were derived, yielding 5 × (6 + 7) = 65 connectivity features per channel. Combined with the nonlinear measures, each channel was represented by 77 features, forming a final feature-based input of (epochs, 19, 77). These engineered representations serve as the sole input to the two models evaluated, EEGNet and a Transformer-based architecture, allowing a focused comparison of how modern neural networks perform when trained exclusively on structured entropy- and graph-based features.

#### 2.3.1 Nonlinear Dynamical Measures

The brain constitutes a highly complex and inherently nonlinear system, in which neural interactions and oscillatory processes exhibit dynamics that cannot be adequately described by linear assumptions. Electroencephalography (EEG) signals, reflecting large-scale neural activity, naturally embody these nonlinear dynamics arising from the coupling of neuronal populations and the presence of feedback mechanisms across multiple temporal and spatial scales [38, 39]. Consequently, linear analytical approaches may fail to capture essential information related to the irregular, nonstationary, and complex characteristics of brain activity. To overcome this limitation, nonlinear dynamical measures have been increasingly adopted in EEG analysis to quantify complex temporal fluctuations and to detect subtle alterations associated with cognitive processes and pathological conditions [40, 41]. These measures offer a robust framework for characterizing the intrinsic complexity, irregularity, and potential chaotic behavior of EEG signals, thereby providing deeper insights into functional brain states and disease-related changes compared to conventional linear features.

In this study, we extracted a comprehensive set of nonlinear features using the NeuroKit2, nolds, and AntroPy Python libraries, which provide reliable implementations for entropy-, fractal-, and chaos-based analyses of physiological time series. These toolboxes facilitate reproducible and standardized computation of nonlinear features, ensuring methodological consistency across subjects and experimental conditions.

##### Approximate Entropy (ApEn):

Quantifies the unpredictability of a time series. Higher ApEn indicates more irregular and complex brain activity, while lower values reflect more regular dynamics, often seen in deep sleep or pathological conditions.

$$\text{ApEn}(m,r,N)=\Phi^{m}(r)-\Phi^{m+1}(r)$$

where $\Phi^{m}(r)=\frac{1}{N-m+1}\sum_{i=1}^{N-m+1}\;\text{ln}\;C_{i}^{m}(r)$ and $C_{i}^{m}(r)$ is the fraction of patterns similar to the i-th pattern within tolerance r.

##### Sample Entropy (SampEn):

Measures signal regularity by estimating the probability that similar patterns repeat.

$$\text{SampEn}(m,r,N)=-\text{ln}\frac{A}{B}$$

where A is the number of matching template pairs of length m+1, and B is the number of matching template pairs of length m.

##### Multiscale Entropy (MSE):

Extends entropy analysis across multiple temporal scales.

$$MSE(\tau )=\text{SampEn}\left(x^{\left(\tau \right)},m,r\right)$$

where $x^{\left(\tau \right)}$ is the coarse-grained time series at scale factor τ.

##### Fuzzy Entropy (FuzzyEn):

Based on fuzzy membership functions, defined as:

$$\text{FuzzyEn}(m,r,n)=\text{ln}\frac{\sum_{i=1}^{N-m}D_{i}^{m}}{\sum_{i=1}^{N-m}D_{i}^{m+1}}$$

where $D_{i}^{m}=\frac{1}{N-m-1}\sum_{j\neq i}e^{-{\left[d_{ij}^{m}\right]}^{n}/r}$.

##### Kolmogorov–Sinai Entropy (K–S Entropy):

Quantifies the average rate of information production:

$$h_{KS}=\sum_{i=1}^{M}\lambda_{i}^{+}$$

where $\lambda_{i}^{+}$ are the positive Lyapunov exponents.

##### Correlation Dimension (CD):

Estimates fractal dimension:

$$D_{2}=\underset{r\to 0}{\text{lim}}\frac{\text{ln}\;C(r)}{\text{ln}\;r}$$

with $C(r)={\text{lim}}_{N\to \infty }\frac{1}{N^{2}}\sum_{i,j}H\left(r-\left\|x_{i}-x_{j}\right\|\right)$.

##### Detrended Fluctuation Analysis (DFA):

Quantifies long-range correlations:

$$F(s)\propto s^{\alpha }$$

where F(s) is the fluctuation function and α is the scaling exponent.

##### Katz Fractal Dimension (Katz FD):


$$FD_{\text{Katz}}=\frac{{\text{log}}_{10}(n)}{{\text{log}}_{10}(n)+{\text{log}}_{10}\left(\frac{d}{L}\right)}$$

where n is the number of points, d the diameter, and L the curve length.

##### Higuchi Fractal Dimension (Higuchi FD):


$$FD_{\text{Higuchi}}=\frac{\text{ln}\;L(k)}{\text{ln}(1/k)}$$

where L(k) is the average length of the curve at scale k.

##### Hurst Exponent (H):

Captures long-term memory:

$$F(T)=cT^{H}$$

where F(T) is the rescaled range of the cumulative deviation of the signal and H is the Hurst exponent.

##### Lyapunov Exponent (LE):

Measures sensitivity to initial conditions:

$$\lambda =\underset{t\to \infty }{\text{lim}}\frac{1}{t}\;\text{ln}\;\frac{\left|\delta x(t)\right|}{\left|\delta x(0)\right|}$$

where δx(t) is the divergence between two initially close trajectories.

##### Lempel–Ziv Complexity (LZC):

Quantifies distinct patterns in the binary sequence representation:

$$C_{LZ}=\frac{c(n)}{n/{\text{log}}_{2}n}$$

where c(n) is the number of distinct substrings and n the sequence length.

Together, these nonlinear measures offer complementary perspectives on EEG complexity, allowing for a multidimensional characterization of neural activity across different states and conditions.

#### 2.3.2 Connectivity Matrix and Graph Theory

In addition to the analysis of temporal dynamics, functional interactions between brain regions were examined using frequency-domain connectivity measures computed with the MNE-Python toolbox. Specifically, spectral connectivity was estimated using the mne_connectivity.spectral_connectivity_time function with the coherence method (method=’coh’) [42]. This approach quantifies coherence between pairs of EEG channels, yielding connectivity matrices that represent the strength of frequency-specific interactions. Coherence is a well-established and robust measure of neural synchronization that captures both amplitude and phase relationships, making it particularly suitable for assessing oscillatory coupling and functional connectivity in EEG signals [43–45].

Rather than being used directly as features, the resulting connectivity matrices were employed to construct graph-based representations of brain networks, from which a range of graph-theoretical metrics were extracted to characterize both local and global network properties. Graph-based features have been extensively utilized in neuroscience research to study cognitive functions, brain disorders, and network efficiency [46–49]. Graph-theoretical measures provide a principled quantitative framework for describing the topological organization of functional brain networks derived from EEG data, enabling the investigation of how neural systems support both segregated and integrated information processing. Furthermore, these measures facilitate the identification of alterations in network organization associated with different cognitive states and pathological conditions [46, 50].

In this study, the NetworkX library was used to compute a comprehensive set of local and global graph-theoretical metrics to assess the organization and efficiency of the reconstructed brain networks.

##### Graph-Theoretic Local (Node-level) Features

Local Features provide a characterization of the role and properties of each individual node within the network.

Let G=(V,E) denote an undirected weighted graph, where V is the set of N=|V| nodes and E is the set of edges. The adjacency matrix is $A=\left[a_{ij}\right]$, where $a_{ij}=1$ if an edge exists between nodes i and j, and 0 otherwise. The weighted adjacency matrix is $W=\left[w_{ij}\right]$, where $w_{ij}\geq 0$ denotes the connection weight between nodes i and j.

###### Degree (Unweighted):

The number of direct connections for each node, representing the most fundamental measure of a node’s connectivity.


$$k_{i}=\sum_{j}a_{ij}$$

###### Strength (Weighted Degree):

The sum of the weights of all connections for a node, reflecting the total functional influence or activity level of a brain region.


$$s_{i}=\sum_{j}w_{ij}$$

###### Clustering Coefficient (Weighted):

Measures the extent to which a node’s neighbors are interconnected, taking connection weights into account. It reflects the local specialization and segregation of information processing around a node [51].


$$C_{i}^{\left(w\right)}=\frac{1}{k_{i}\left(k_{i}-1\right)}\sum_{j,k}{\left({\overset{ˆ}{w}}_{ij}{\overset{ˆ}{w}}_{jk}{\overset{ˆ}{w}}_{ki}\right)}^{1/3},\;\text{where}\;{\overset{ˆ}{w}}_{ij}=\frac{w_{ij}}{\text{max}\left(w_{pq}\right)}$$

###### Betweenness Centrality (Weighted):

Quantifies how often a node acts as a bridge along the shortest weighted path between other nodes, helping to identify critical hubs for information flow.

$$BC_{i}=\sum_{j\neq k\neq i}\frac{\sigma_{jk}(i)}{\sigma_{jk}},\;{\tilde{BC}}_{i}=\frac{2\;BC_{i}}{\left(N-1)(N-2\right)}$$

where $\sigma_{jk}$ is the number of shortest paths between nodes j and k, and $\sigma_{jk}(i)$ is the number of those paths that pass through node i.

###### Eigenvector Centrality (Weighted):

A measure of a node’s influence based on its connections to other influential nodes, considering connection weights.

$$\lambda v_{i}=\sum_{j}w_{ij}v_{j},$$

where v is the principal eigenvector of W and λ is the corresponding largest eigenvalue.

###### Local Efficiency:

Represents the efficiency of information transfer within the immediate neighborhood of a node, indicating the resilience of the local subnetwork to damage.

$$E_{\text{loc}}(i)=\frac{1}{k_{i}\left(k_{i}-1\right)}\sum_{\begin{array}{c} j,k\in G_{i}\\ j\neq k \end{array}}\frac{1}{d_{jk}^{\left(i\right)}},$$

where $d_{jk}^{\left(i\right)}$ is the shortest path length between nodes j and k in the subgraph $G_{i}$ formed by the neighbors of node i.

##### Graph-Theoretic Global (Graph-level) Features

Global features provide a single value describing the overall topology and organization of the entire brain network.

###### Global Efficiency:

Reflects the overall efficiency of parallel information transfer and integration across the entire network [52].

$$E_{\text{glob}}=\frac{1}{N(N-1)}\sum_{i\neq j}\frac{1}{d_{ij}},$$

where $d_{ij}$ is the shortest path distance between nodes i and j.

###### Characteristic Path Length (Weighted):

The average shortest path length between all pairs of nodes, considering connection weights. Lower values indicate more efficient communication across the brain network.


$$L=\frac{1}{\frac{N(N-1)}{2}}\sum_{i<j}d_{ij}$$

###### Average Clustering Coefficient:

The average of the weighted clustering coefficients of all nodes, providing a global measure of the network’s tendency for specialized, local processing.


$$\overset{‾}{C}=\frac{1}{N}\sum_{i=1}^{N}C_{i}^{\left(w\right)}$$

###### Small-worldness:

Characterizes networks that have both dense local clustering and short global path lengths [53]. This property is thought to enable a balance between segregated and integrated information processing.

$$\sigma =\frac{C/C_{\text{rand}}}{L/L_{\text{rand}}}$$

where $C_{\text{rand}}$ and $L_{\text{rand}}$ denote the average clustering coefficient and path length of comparable random networks.

###### Assortativity Coefficient:

Measures the tendency of nodes to connect to other nodes with similar degrees, reflecting the presence of a central core of high-degree hubs [54].

$$r=\frac{\sum_{(i,j)\in E}\left(k_{i}-\mu \right)\left(k_{j}-\mu \right)}{\sum_{(i,j)\in E}{\left(k_{i}-\mu \right)}^{2}},\;\text{where}\;\mu =\frac{1}{2M}\sum_{(i,j)\in E}\left(k_{i}+k_{j}\right)$$

and M=|E| is the total number of edges.

###### Network Density:

Represents the ratio of actual connections to the total possible connections in the network.


$$\rho =\frac{2|E|}{N(N-1)}$$

###### Transitivity:

A global version of the clustering coefficient, reflecting the overall network cohesiveness and propensity for clique formation.


$$T=\frac{3\times \text{number}\;\text{of}\;\text{triangles}}{\text{number}\;\text{of}\;\text{connected}\;\text{triplets}}=\frac{\sum_{i}\sum_{j<k}a_{ij}a_{ik}a_{jk}}{\sum_{i}k_{i}\left(k_{i}-1\right)}$$

###### Notes:

- Weighted shortest paths are computed from edge lengths $l_{ij}=1/w_{ij}$ (assuming higher weights indicate stronger connections).
- If the network is disconnected, efficiency measures remain valid since $1/d_{ij}=0$ for disconnected pairs, but L is often computed on the largest connected component.
- All measures were computed using the NetworkX library with the weight=’weight’ parameter.

### 2.4 Model Architectures

**Fig. 1:**
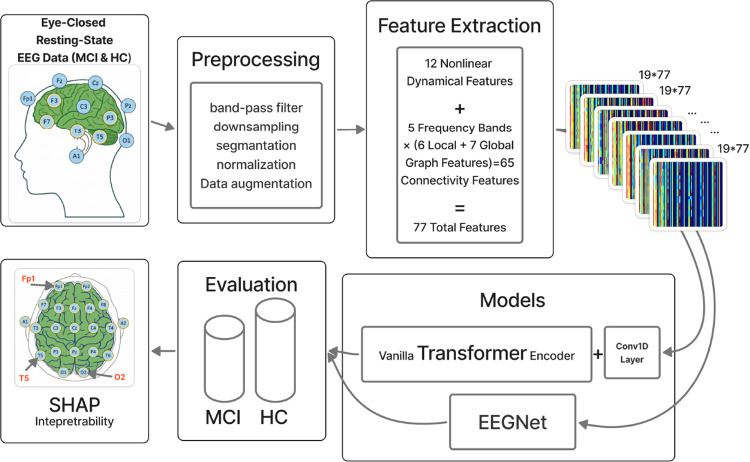
Overall experimental pipeline. Eyes-closed resting-state EEG from MCI and HC participants was preprocessed and used for feature extraction, including nonlinear entropy measures and graph-theoretic metrics. Two model types were evaluated using these features: EEGNet and the proposed Transformer architecture. SHAP-based model explanations were computed exclusively for the feature-based Transformer.

As illustrated in Figure 1, this study adopts a fully feature-driven EEG classification framework and evaluates two deep learning architectures trained on an identical engineered representation: EEGNet and a Transformer-based model. Both networks receive the same structured input composed of entropy-based measures, nonlinear dynamical descriptors, and graph-theoretic connectivity features, enabling a controlled comparison that isolates architectural modeling capacity from input representation. EEGNet is employed as a compact convolutional baseline and is adapted from its original raw-EEG formulation to operate on the extracted feature matrices. Although EEGNet was originally designed to learn spatial-temporal filters directly from time-domain EEG signals, in this work it processes feature-channel representations to assess the effectiveness of convolutional inductive biases when applied to structured neurophysiological descriptors. The primary model proposed in this study is a feature-based Transformer implemented using a *vanilla Transformer encoder* architecture based entirely on self-attention mechanisms [55]. Unlike hybrid or task-specific Transformer variants, this model follows the standard encoder design, allowing direct modeling of interactions across EEG channels and feature dimensions without convolutional or recurrent dependencies.

The network begins with a one-dimensional convolutional embedding layer that projects the input feature matrix into a higher-dimensional latent space while capturing short-range dependencies. To preserve ordering information, a trainable positional embedding is added to the embedded representation. The model then applies a stack of N=3 Transformer encoder blocks, each consisting of a multi-head self-attention layer with H=4 attention heads followed by a position-wise feed-forward network. Residual connections and layer normalization are applied around each sub-layer to stabilize training and support deeper representations. To reduce overfitting, several regularization strategies are employed, including dropout within the attention and feed-forward layers, $L_{2}$ weight decay on trainable parameters, and batch normalization following the convolutional embedding. The resulting representations are aggregated using global average pooling and passed through fully connected layers before a softmax output layer produces the final classification. By leveraging self-attention over structured entropy-, nonlinear dynamical-, and graph-derived EEG features, the Transformer architecture captures complex cross-channel and cross-feature dependencies that are difficult to model using convolutional filters alone. This design enables effective integration of information-theoretic complexity, dynamical behavior, and functional network organization within a standard and interpretable Transformer encoder framework.

All experiments were conducted in the Kaggle cloud environment using an NVIDIA Tesla P100 GPU (16 GB VRAM) and 30 GB of system memory. Training time differed between the two network architectures: the Transformer required longer training due to its deeper structure and multi-head self-attention mechanism, whereas EEGNet converged more rapidly owing to its compact convolutional design. Although the preprocessing pipeline for computing nonlinear dynamical features and graph-theoretic measures was relatively time-consuming, once these features were extracted, both network training and inference became substantially faster. Despite the additional computational cost incurred during feature extraction, this feature-driven approach enables the simultaneous achievement of high classification accuracy and improved interpretability.

### 2.5 Experimental Setup

All experiments were performed in Python using TensorFlow and Keras within a unified training framework to ensure a fair comparison between the two architectures. Since this study adopts a fully feature-driven approach, both models were trained exclusively on the engineered feature representation, which combines nonlinear dynamical measures and graph-theoretical connectivity metrics. The resulting input format is a structured matrix of size 19 × 77, corresponding to 19 EEG channels and 77 features per channel.

#### 2.5.1 Training, Validation, and Testing Procedure

For each network architecture (see Table 2), model training was conducted using the Adam optimizer, categorical cross-entropy loss, and early stopping with patience of 10 epochs to prevent overfitting. Learning rate scheduling was applied following an exponential decay scheme. Training was limited to a maximum of 50 epochs, and the best model weights were preserved via checkpointing based on the highest validation accuracy.

- **EEGNet:** This compact convolutional neural network exploited depthwise and separable convolutions for efficient spatial-temporal feature extraction. The model used a dropout rate of 0.4 and batch size of 4. Learning rate was set to 1 × 10^−5^.
- **Transformer Network:** For the feature representation (19, 77), the Transformer model was composed of six Transformer blocks, each with five attention heads and a feed-forward dimension of 64. A convolutional embedding layer with 128 filters (kernel size = 5) was used to project the input features into the embedding space. Regularization included a dropout rate of 0.1 and an $L_{2}$ penalty of 0.0005. The model was trained for 50 epochs with a learning rate of 1 × 10^−4^ and a batch size of 8.

All two networks were trained and validated using identical callbacks, including early stopping, model checkpointing, and learning rate scheduling, to ensure reproducibility and stability of results.

#### 2.5.2 Evaluation Metrics

Performance evaluation employed a set of complementary metrics to assess both overall classification accuracy and class-specific sensitivity. The primary metrics were accuracy, precision, recall, F1-score, and the area under the ROC curve (AUC).

$$\text{Accuracy}=\frac{TP+TN}{TP+TN+FP+FN},$$

where TP,TN,FP, and FN denote true positives, true negatives, false positives, and false negatives, respectively.

Precision and recall were calculated as:

$$\text{Precision}=\frac{TP}{TP+FP},\;\text{Recall}=\frac{TP}{TP+FN}.$$

The F1-score was computed as the harmonic mean of precision and recall:

$$\text{F1-score}=2\times \frac{\text{Precision}\times \text{Recall}}{\text{Precision}+\text{Recall}}.$$

Macro-averaged F1-scores were reported to give equal weight to both classes (MCI and HC).

The AUC metric quantifies the model’s discriminative ability across all possible decision thresholds, providing a robust measure of separability between MCI and HC.

All metrics were computed on the held-out test set using the model checkpoint with the highest validation accuracy. Results are reported as mean ± standard deviation across validation folds, and confusion matrices were generated to visualize classification tendencies.

#### 2.5.3 Between-Subject Cross-Validation Method

To ensure subject-independent evaluation and prevent data leakage across participants, a group-based data partitioning strategy was employed using GroupShuffleSplit. This approach enforces strict subject-level separation, ensuring that EEG samples from the same individual are assigned exclusively to a single data partition (training, validation, or testing). As described in the preprocessing stage, offline data augmentation was applied in a subject-preserving manner, and all subsequent data partitioning was performed using strict group-based splitting. As a result, the evaluation reflects a realistic cross-subject scenario, assessing the model’s ability to generalize to previously unseen individuals rather than exploiting subject-specific patterns.

In the outer partitioning step, a single group-based split divided the dataset into training (80%) and testing (20%) subsets according to subject identifiers. The test set was completely held out during model development and used solely for final performance evaluation. Within the training subset, repeated group-based splits were applied to further divide the data into training and validation sets for model selection, early stopping, and learning rate scheduling. Model checkpoints were saved based on the best validation accuracy in each split to ensure stable and reproducible training. This evaluation framework preserves strict subject-level independence across all data partitions and enables a fair comparison between different model architectures, including EEGNet and Transformer-based models, under identical data-splitting conditions.

### 2.6 Model Interpretability with SHAP

To elucidate the factors driving the predictions of the proposed feature-based Transformer model, we employed SHAP, a model-agnostic framework for interpreting complex machine learning models. SHAP enables both local and global attribution of prediction outcomes by quantifying the contribution of each input feature to the model’s output. Prior to model training and SHAP analysis, all extracted features were normalized to ensure comparable scaling and to facilitate stable and meaningful learning. Specifically, normalization was performed independently for each feature across all epochs and all EEG channels. This feature-wise normalization prevents dominance of features with larger numerical ranges and enables the deep learning model to discover new and meaningful relationships among features, channels, and epochs based on their relative variations rather than absolute magnitudes.

SHAP analysis was conducted exclusively on the feature-based Transformer model using the SHAP library, following a consistent procedure across cross-validation folds:

- **Explainer Initialization:** A GradientExplainer, suitable for smooth approximations in deep neural networks, was used to compute SHAP values. For each cross-validation fold, a background reference set of up to 300 randomly selected training samples (limited by fold size) was employed to stabilize the estimation of the model’s baseline behavior.
- **SHAP Value Computation:** For each of the five cross-validation folds, SHAP values were computed for 200 samples drawn from the corresponding held-out test set, enabling fold-specific interpretability while preserving subject independence.
- **Aggregation and Global Importance:** The absolute SHAP values were averaged across all evaluated test samples and across all five folds. This aggregation yielded robust global importance scores for individual features and EEG channels, providing a comprehensive and stable representation of the patterns learned by the Transformer model.

This analysis framework allows direct interpretation of how normalized nonlinear dynamical features and graph-theoretic connectivity measures jointly influence model decisions, thereby linking the Transformer’s predictions to physiologically meaningful EEG characteristics.

## 3 Results

**Fig. 2:**
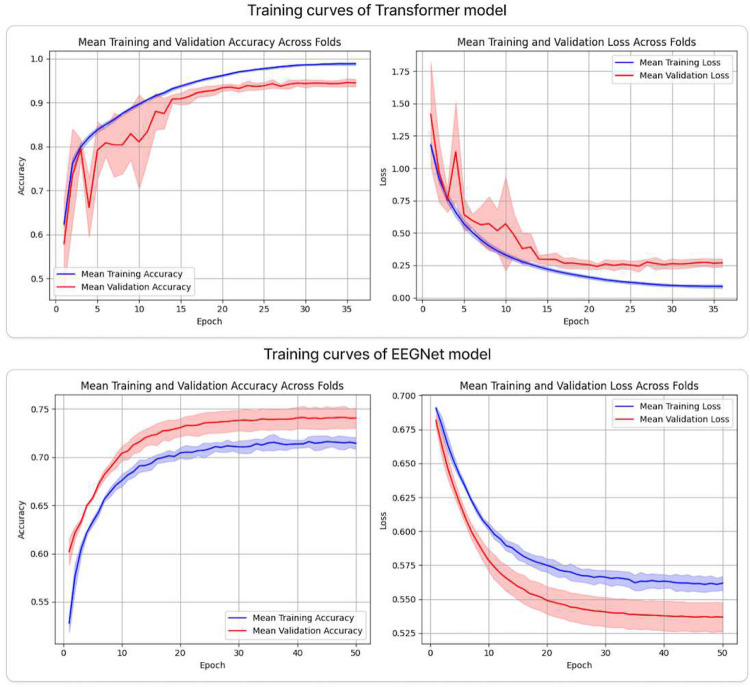
This figure illustrates the mean training and validation accuracy and loss for the Transformer and EEGNet models, evaluated using 5-fold cross-validation. For each model, accuracy and loss are plotted across training epochs. The solid blue and red curves denote the mean training and validation metrics, respectively, and the shaded areas represent the standard deviation across folds. Overall, the Transformer model attains higher accuracy compared to EEGNet.

Table 3 presents the average classification performance (mean ± standard deviation) of the two models on the held-out test set. Figure 2 complements these results by showing the mean training and validation accuracy and loss for the Transformer and EEGNet architectures, computed using 5-fold cross-validation.

**Fig. 3:**
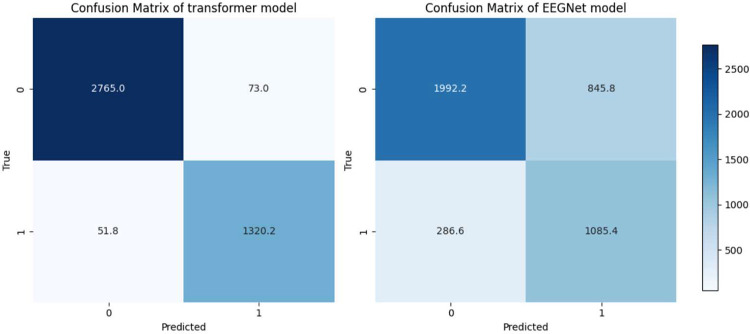
Average confusion matrices from 5-fold cross-validation. This figure compares the performance of the two models, Transformer and EEGNet, on the binary classification task, with each matrix representing the results averaged across the validation folds.

Among the two evaluated architectures, the proposed feature-based Transformer model demonstrated substantially superior performance across all evaluation metrics. It achieved an accuracy of 97.04%±0.72, an AUC of 0.9934±0.0019, and an F1-score of 0.9664±0.0081, indicating highly reliable and robust classification. In contrast, EEGNet showed markedly lower performance, with an accuracy of 73.10%±0.59, an AUC of 0.8228±0.0130, and an F1-score of 0.7179±0.0061. The observed performance gap across all metrics highlights the clear advantage of the Transformer-based approach for the task under investigation. These differences are further reflected in the confusion matrices (Figure 3), where the Transformer demonstrates clear class separability with minimal misclassification, whereas EEGNet shows notably higher error rates. Consistent improvements in precision and recall for the Transformer indicate more balanced and dependable decision-making across classes. Overall, the results confirm a strong and comprehensive advantage of the proposed Transformer architecture over EEGNet on the test data.

**Fig. 4:**
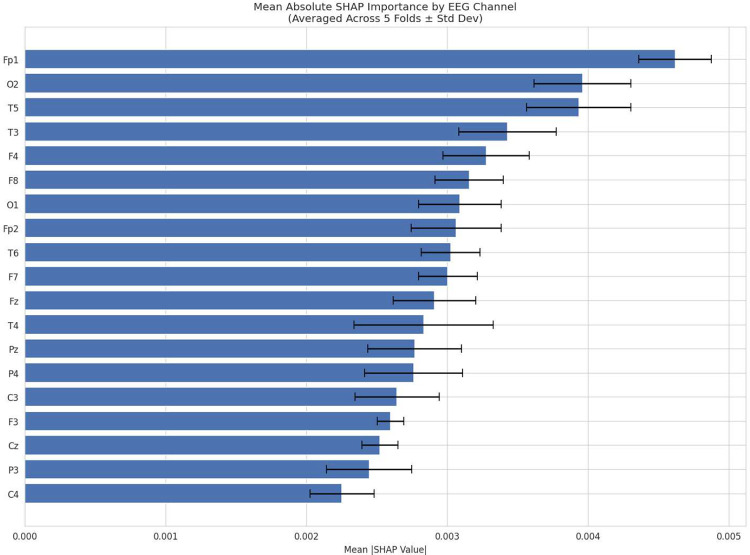
Average channel importance (mean absolute SHAP values) across all features and 5-fold cross-validation. The frontal pole channel Fp1 exhibits the highest importance, followed closely by the right occipital channel O2 and the left posterior temporal channel T5, highlighting substantial contributions from frontal, occipital, and temporal regions to the model’s MCI classification decisions.

**Fig. 5:**
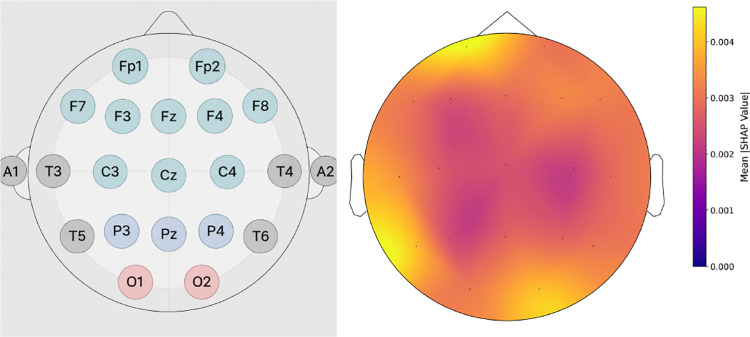
Topographical distribution of average absolute SHAP values across all features and folds. Higher importance is observed in frontal (Fp1), occipital (O2), and temporal (T5) regions, reflecting a distributed spatial pattern of model reliance rather than a single dominant cortical area. Yellow tones indicate greater contribution to the Transformer’s classification decisions.

**Fig. 6:**
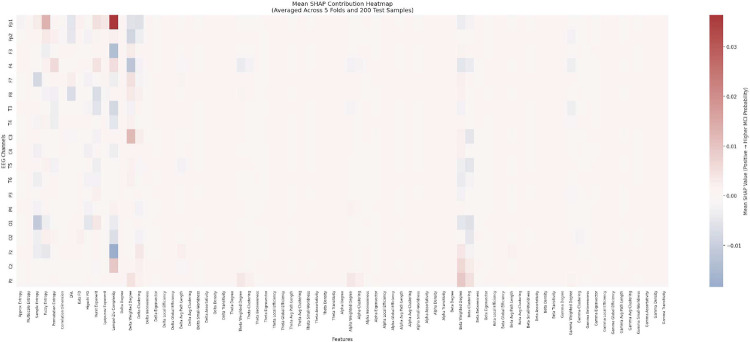
Averaged SHAP heatmap illustrating channel–feature interactions across all samples and folds. Rows correspond to EEG channels, while columns represent individual nonlinear and connectivity features. Red and blue values denote positive and negative contributions to the model output, respectively, highlighting heterogeneous feature relevance across channels.

**Fig. 7:**
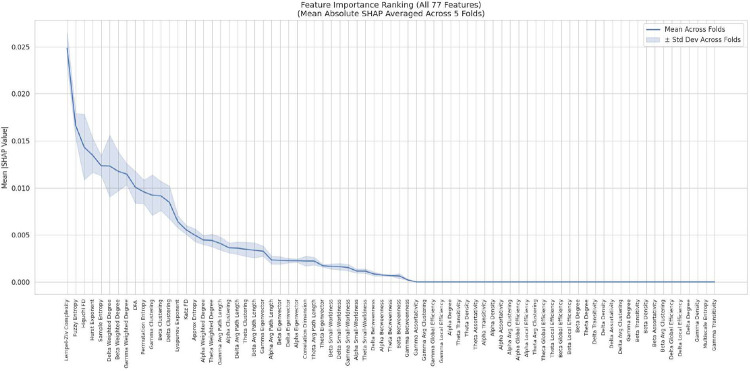
Global feature importance measured by mean absolute SHAP values across 5-fold cross-validation. Nonlinear dynamical measures dominate the top ranks, with Lempel–Ziv Complexity, Fuzzy Entropy, Higuchi Fractal Dimension, and the Hurst Exponent showing the highest importance. Connectivity features contribute subsequently, with weighted-degree and clustering metrics appearing prominently across multiple frequency bands.

**Fig. 8:**
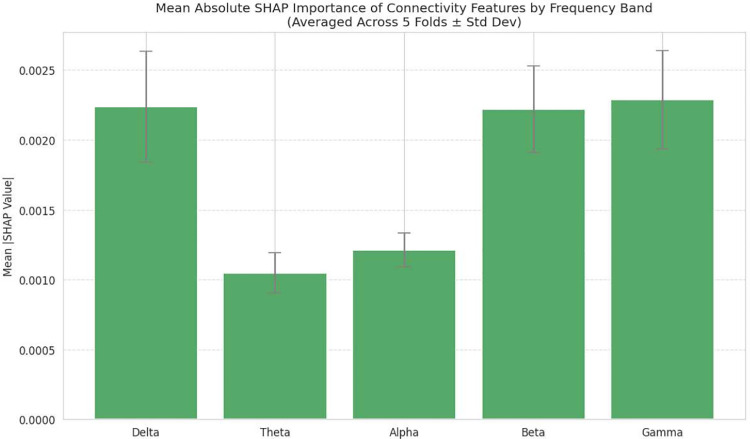
Relative importance of connectivity features by frequency band, computed as the sum of absolute SHAP values and averaged across folds. Connectivity in the Gamma band exhibits the highest contribution, followed by Delta and Beta bands, whereas Alpha and Theta bands show comparatively lower influence on the model’s predictions.

To gain insight into the model’s decision-making process, we analyzed SHAP values derived from the feature-based Transformer model. The results reveal a structured pattern of importance across EEG channels, feature types, and frequency bands. As reported in Table 4 and illustrated in Figure 4, the frontal pole channel Fp1 exhibited the highest average SHAP importance across folds. This was followed by the left temporal channel T5 and the occipital channel O2, indicating that frontal, temporal, and posterior brain regions all contribute meaningfully to the classification task. Overall, the distribution of channel importances suggests that the model leverages information from multiple cortical regions rather than relying on a single localized source. Figures 5 and 6 further illustrate these findings by depicting the spatial distribution of SHAP values across the scalp and the interaction between channels and feature-level contributions. Heatmap visualizations preserve the signed SHAP values to highlight the direction of each feature’s contribution, whereas magnitude-based summaries use absolute SHAP values to quantify relative importance (Figure 6 and 7). At the feature level, non-linear dynamical measures were consistently among the most influential. As shown in Table 4 and Figure 7, Lempel–Ziv Complexity achieved the highest average SHAP value, followed by Fuzzy Entropy, Higuchi Fractal Dimension, and the Hurst Exponent. These results indicate that signal complexity, irregularity, and long-range temporal dynamics play a central role in the model’s predictions. Sample Entropy also contributed to the classification performance, albeit with lower average importance compared to the top-ranked complexity measures. Regarding connectivity features, the analysis by frequency band (Table 4 and Figure 8) shows that connectivity in the Gamma band was most influential, followed closely by the Beta and Delta bands. In contrast, connectivity in the Alpha and Theta bands exhibited substantially lower importance. This pattern suggests that both high-frequency and slow-wave connectivity dynamics provide discriminative information for the model.

## 4 Discussion

The comparative evaluation of the feature-based Transformer and EEGNet architectures provides clear insight into how model inductive biases interact with structured, engineered EEG representations in the context of Mild Cognitive Impairment (MCI) classification. Using a unified input composed of entropy-based, nonlinear dynamical, and graph-theoretic features, the Transformer consistently outperformed the adapted EEGNet baseline. This performance advantage can be attributed to the multi-head self-attention mechanism, which is inherently suited for modeling long-range dependencies and cross-feature interactions within heterogeneous feature spaces. In contrast, EEGNet relies on convolutional filters with localized receptive fields, which, although effective for extracting spatial-temporal patterns from raw EEG, are less capable of capturing global and relational dependencies within structured feature matrices. The engineered 77-dimensional feature representation, consisting of 12 nonlinear dynamical measures and 65 graph-theoretic connectivity metrics extracted across five canonical frequency bands, provided a compact yet biologically grounded summary of brain activity. By jointly encoding signal irregularity, dynamical complexity, and large-scale functional organization, this feature set enabled both models to operate on a richly informative input space without direct access to raw temporal waveforms. The strong performance of the Transformer indicates that attention-based architectures are particularly effective when provided with structured descriptors that explicitly summarize key properties of neural dynamics and network topology, especially under strict cross-subject evaluation protocols.

A major strength of the proposed framework lies in its interpretability. Because each input dimension corresponds to a well-defined physiological or network-level construct, SHAP-based analysis of the Transformer model enabled direct identification of the features, frequency bands, and EEG channels most responsible for classification decisions. This level of interpretability is difficult to achieve in end-to-end models trained on raw EEG signals, where learned filters often lack clear neurophysiological correspondence. Although the feature-adapted EEGNet achieved competitive performance as a baseline, SHAP analysis was restricted to the Transformer model, whose higher classification accuracy supports more reliable and interpretable attribution results. The superior performance of the Transformer therefore provides a more dependable basis for SHAP-based interpretation. SHAP values are designed to be additive for individual predictions: for a single sample, the sum of SHAP values across all features exactly equals the difference between the model’s prediction and the expected (baseline) output. However, this additivity does not extend to aggregations over subsets of features, channels, frequency bands, or when using absolute values. As a result, the summed SHAP values presented in our plots do not correspond to any specific model output and are not required to equal a particular fixed value. Rather, they function as relative measures of contribution magnitude, enabling meaningful comparisons of the influence exerted by different channels, features, or frequency bands on the model’s decisions. From a computational perspective, the Transformer required greater memory usage and longer training times than EEGNet due to its deeper architecture and self-attention operations. However, once entropy-based, nonlinear dynamical, and graph-theoretic features were extracted, both training and inference were computationally efficient and substantially faster than conventional raw-EEG deep learning pipelines. This trade-off highlights the practical utility of feature-based modeling: while feature extraction introduces an upfront computational cost, it is performed only once, after which the framework supports rapid, scalable, and reproducible model evaluation.

Beyond overall classification performance, SHAP analysis offered insight into the neurophysiological alterations associated with Mild Cognitive Impairment by revealing a coherent pattern across multiple nonlinear dynamical and connectivity-based features. Among all extracted descriptors, Lempel–Ziv Complexity (LZC) showed the highest SHAP importance, indicating that reductions in algorithmic signal complexity are a primary contributor to the differentiation between MCI and healthy aging. Decreased LZC has been linked to impaired information encoding capacity and reduced neural variability, both of which are considered hallmark features of early neurodegenerative processes [56, 57]. Notably, LZC was closely followed by other nonlinear measures, including Fuzzy Entropy, Higuchi Fractal Dimension, Hurst Exponent, Sample Entropy, and Detrended Fluctuation Analysis. The comparable SHAP values of these metrics suggest that MCI-related EEG alterations manifest as a global reorganization of temporal dynamics rather than isolated changes in a single complexity index. Previous work has demonstrated that entropy- and fractal-based measures are particularly sensitive to early disruptions in long-range temporal correlations and self-similarity of neural signals, making them effective markers for detecting prodromal cognitive decline before pronounced spectral abnormalities emerge [58, 59]. In addition to dynamical features, graph-theoretic connectivity metrics, particularly weighted degree and clustering coefficients in Delta, Beta, and Gamma bands, also exhibited meaningful SHAP contributions. This pattern suggests that MCI is characterized by concurrent degradation of local signal dynamics and large-scale functional network organization. Altered balance between functional integration and segregation has been consistently reported in MCI, reflecting early breakdown of efficient information transfer across distributed brain networks [60, 61]. Collectively, these findings support a multiscale view of early cognitive impairment, in which reductions in neural complexity are tightly coupled with disruptions in functional connectivity. Neural oscillations across distinct frequency bands such as gamma, beta and delta have been increasingly investigated as potential electrophysiological biomarkers of mild cognitive impairment (MCI). Gamma-band activity, which supports high-level cognitive processes including memory and attention, shows altered patterns in cognitive impairment; early evidence suggests that MCI patients may exhibit differences in gamma power and functional connectivity relative to healthy controls, reflecting disrupted cortical synchronization underlying cognitive processing, and interventions modulating gamma rhythms have been proposed to improve network integration in neurodegenerative conditions [62]. Beta oscillations, often associated with sensorimotor integration and top-down cognitive control, demonstrate alterations in power and connectivity in MCI populations, with beta-theta ratios differing between MCI and healthy aging in spectral periodic analyses, indicating shifting balance between faster and slower rhythms in early cognitive decline [63]. Additionally, delta oscillations, linked to large-scale neural coordination, are more prominent in MCI and reflect impaired network efficiency, particularly in resting-state functional networks potentially linked to amyloid pathology [64]. Collectively, these frequency-specific changes in neural rhythms suggest a progressive slowing and dysregulation of brain dynamics in MCI, offering promising avenues for early detection and intervention via EEG biomarkers [65]. At the channel level, SHAP analysis revealed a distributed pattern of importance, with the frontal pole electrode Fp1 exhibiting the highest contribution, followed by the left temporal channel T5 and the occipital channel O2. This ranking indicates that frontal, temporal, and posterior cortical regions jointly contribute to the discrimination between MCI and healthy aging, rather than the model relying on a single focal source. The prominence of Fp1 highlights the role of prefrontal dysfunction in early cognitive decline, as this region is critically involved in executive control, working memory, and strategic components of episodic retrieval [66, 67]. Reductions in frontal signal complexity and disrupted prefrontal connectivity have been consistently reported in prodromal Alzheimer’s disease and MCI, reflecting early executive dysfunction and impaired top-down regulation [68]. The high importance of the left temporal electrode T5 underscores the contribution of temporal-lobe dysfunction, which is closely linked to memory encoding and retrieval processes. Temporal regions are among the earliest affected structures in amnestic MCI, and alterations in temporal EEG dynamics and connectivity have been widely reported as electrophysiological correlates of memory impairment and medial temporal lobe vulnerability. The occipital channel O2, ranking third in importance, indexes activity in the right occipital cortex, which supports early visual processing and posterior alpha rhythm generation. Alterations in occipital oscillations, including attenuated alpha power and disrupted posterior-to-frontal connectivity, are well-documented EEG markers of MCI and early Alzheimer’s disease [69, 70]. Collectively, this spatial distribution of channel importance aligns with network-based models of early neurodegeneration that emphasize progressive disintegration of long-range cortico-cortical communication.

Repetitive transcranial magnetic stimulation (rTMS) has emerged as a non-invasive neuromodulatory intervention for cognitive enhancement in individuals with MCI, targeting cortical networks involved in memory and executive functions. Common protocols typically employ high-frequency (10–20 Hz) stimulation over the left dorsolateral prefrontal cortex (DLPFC) or bilateral prefrontal regions, with session durations ranging from 10 to 30 minutes across multiple days or weeks [71, 72]. Previous studies suggest that rTMS may enhance cortical excitability and functional connectivity, leading to improvements in neuropsychological outcomes such as working memory, attention, and global cognition in MCI populations [73]. Notably, the EEG-derived biomarkers identified in the present study, particularly alterations in gamma and beta band connectivity, are consistent with frequency ranges and cortical networks reported to be influenced by rTMS protocols. Although causal or mechanistic inferences cannot be drawn from the current findings, this convergence suggests that the neural patterns captured by our Transformer-based feature-driven model may reflect network dynamics that are also targeted by neuromodulatory interventions. From this perspective, rTMS could represent a complementary therapeutic approach, while EEG-based models such as ours may offer objective biomarkers for characterizing network-level dysfunction and monitoring intervention-related changes in future longitudinal or interventional studies.

Taken together, these findings indicate that integrating engineered neurophysiological features with Transformer-based learning yields a powerful, interpretable, and computationally practical framework for EEG-based MCI detection. Future extensions of this work may move beyond binary classification toward longitudinal modeling of disease progression, including prediction of conversion from MCI to Alzheimer’s disease. Integrating EEG with complementary modalities such as fNIRS, MRI, or detailed cognitive assessments may further enhance diagnostic sensitivity. In addition, more detailed analyses of frequency-specific contributions, cross-frequency coupling, and individualized network signatures could deepen understanding of the neural mechanisms underlying cognitive decline. Finally, optimization of feature extraction pipelines and exploration of lightweight Transformer variants may facilitate real-time, deployable systems for clinical and home-based cognitive monitoring.

## 5 Conclusion

This study presented a systematic comparison of EEGNet and a Transformer-based architecture within a unified, feature-driven framework for cross-subject classification of Mild Cognitive Impairment. By replacing raw EEG signals with a structured representation composed of entropy-based, nonlinear dynamical, and graph-theoretic features, the proposed approach achieved strong classification performance while preserving neurophysiological interpretability. A strict subject-independent evaluation strategy ensured that reported results reflect genuine generalization to unseen individuals. Across all evaluations, the feature-based Transformer consistently outperformed EEGNet, demonstrating the effectiveness of self-attention mechanisms for integrating heterogeneous neurophysiological descriptors and modeling complex cross-channel relationships. SHAP-based interpretability further revealed that classification decisions were driven by biologically meaningful features, frequency bands, and cortical regions known to be affected in early cognitive decline. These results support the value of combining engineered EEG features with attention-based deep learning to develop accurate, interpretable, and clinically relevant tools for early detection of cognitive impairment.

## Figures and Tables

**Table 1:** Demographic distribution of subjects included in this study.

Group	Male (M)	Female (F)	Total
Overall Subjects	98	85	183
MCI	36	20	56
HC	62	65	127

**Table 2:** Hyperparameter settings for Transformer and EEGNet models

Transformer	
Parameter	Value
Input shape	(19, 77)
Num. transformer blocks	6
Num. heads	5
FF dim	64
Conv filters	128
Conv kernel size	5
Dropout rate	0.1
Epochs	50
Batch size	8
L2 rate	0.0005
Learning rate	1e-4

EEGNet	
Parameter	Value
Input shape	(19, 77)
Dropout rate	0.4
Epochs	50
Batch size	4
Learning rate	1e-5
F1	32
D	4
F2	128

**Table 3:** Average performance (mean ± std) of models on test data. The proposed method is highlighted.

Model	Accuracy	AUC	F1-score	Precision	Recall
EEGNet	0.7310 ± 0.0059	0.8228 ± 0.0130	0.7179 ± 0.0061	0.7182 ± 0.0064	0.7465 ± 0.0075
Transformer^1^	0.9704 ± 0.0072	0.9934 ± 0.0019	0.9664 ± 0.0081	0.9647 ± 0.0097	0.9683 ± 0.0065

1 Compared with the alternative model, the proposed model showed clear overall improvement.

**Table 4:** SHAP importances of EEG channels, frequency bands, and features, (mean |SHAP|), averaged across 200 test samples, up to 300 background samples, and 5 cross-validation folds.

Name	Mean |SHAP|	Std Dev
**Channel Importances**		
Fp1	0.004619	0.000257
O2	0.003960	0.000345
T5	0.003934	0.000371
T3	0.003427	0.000345
F4	0.003276	0.000306
F8	0.003157	0.000242
O1	0.003089	0.000293
Fp2	0.003062	0.000319
T6	0.003024	0.000208
F7	0.003004	0.000208
Fz	0.002910	0.000295
T4	0.002834	0.000494
Pz	0.002769	0.000333
P4	0.002762	0.000348
C3	0.002643	0.000299
F3	0.002596	0.000095
Cz	0.002522	0.000128
P3	0.002446	0.000303
C4	0.002252	0.000229
**Connectivity Importances by Frequency Band (Total)**		
Gamma	0.029750	0.004563
Delta	0.029094	0.005143
Beta	0.028851	0.004053
Alpha	0.015753	0.001569
Theta	0.013622	0.001876
**Top 15 Feature Importances**		
Lempel-Ziv Complexity	0.024834	0.001659
Fuzzy Entropy	0.016637	0.001289
Higuchi FD	0.014329	0.003468
Hurst Exponent	0.013464	0.001807
Sample Entropy	0.012350	0.001062
Delta Weighted Degree	0.012321	0.003270
Beta Weighted Degree	0.011761	0.002070
Gamma Weighted Degree	0.011455	0.001118
DFA	0.010073	0.001693
Permutation Entropy	0.009569	0.001247
Gamma Clustering	0.009224	0.002141
Beta Clustering	0.009156	0.001541
Delta Clustering	0.008455	0.001730
Lyapunov Exponent	0.006392	0.000655
Katz FD	0.005523	0.000463

## Data Availability

Researchers interested in accessing the dataset used in this study can contact shalbaf@sbmu.ac.ir for guidance. Since the data do not belong to us, we cannot directly share it; however, we will assist interested researchers by providing information on how they can follow the necessary procedures, including signing the NIH Data Sharing Policy, to obtain access from the data owners.
